# Amino acid digestibility of heat damaged distillers dried grains with solubles fed to pigs

**DOI:** 10.1186/2049-1891-4-44

**Published:** 2013-11-14

**Authors:** Ferdinando Nielsen Almeida, John Kyaw Htoo, John Thomson, Hans Henrik Stein

**Affiliations:** 1 Department of Animal Sciences, University of Illinois, 1207 W Gregory Dr, Urbana, IL 61801, USA; 2 Evonik Industries AG, Rodenbacher Chaussee 4, Hanau 63457, Germany; 3 Evonik Degussa Corporation, 1701 Barrett Lakes Blvd NW Kennesaw, GA 30144, USA

**Keywords:** Amino acids, Digestibility, Distillers dried grains with solubles, Heat damage

## Abstract

The primary objective of this experiment was to determine the effects of heat treatment on the standardized ileal digestibility (SID) of amino acids (AA) in corn distillers dried grains with solubles (DDGS) fed to growing pigs. The second objective was to develop regression equations that may be used to predict the concentration of SID AA in corn DDGS. A source of corn DDGS was divided into 4 batches that were either not autoclaved or autoclaved at 130°C for 10, 20, or 30 min. Four diets containing DDGS from each of the 4 batches were formulated with DDGS being the only source of AA and CP in the diets. A N-free diet also was formulated and used to determine the basal endogenous losses of CP and AA. Ten growing pigs (initial BW: 53.5 ± 3.9 kg) were surgically equipped with a T-cannula in the distal ileum and allotted to a replicated 5 × 4 Youden square design with 5 diets and 4 periods in each square. The SID of CP decreased linearly (*P* < 0.05) from 77.9% in non-autoclaved DDGS to 72.1, 66.1, and 68.5% in the DDGS samples that were autoclaved for 10, 20, or 30 min, respectively. The SID of lysine was quadratically reduced (*P* < 0.05) from 66.8% in the non-autoclaved DDGS to 54.9, 55.3, and 51.9% in the DDGS autoclaved for 10, 20, or 30 min, respectively. The concentrations of SID Arginine, Histidine, Leucine, Lysine, Methionine, Phenylalanine, or Threonine may be best predicted by equations that include the concentration of acid detergent insoluble N in the model (r^2^ = 0.76, 0.68, 0.67, 0.84, 0.76, 0.73, or 0.54, respectively). The concentrations of SID Isoleucine and Valine were predicted (r^2^ = 0.58 and 0.54, respectively) by the Lysine:CP ratio, whereas the concentration of SID Tryptophan was predicted (r^2^ = 0.70) by the analyzed concentration of Tryptophan in DDGS. In conclusion, the SID of AA is decreased as a result of heat damage and the concentration of SID AA in heat-damaged DDGS may be predicted by regression equations developed in this experiment.

## Background

Production of corn distillers dried grains with solubles (DDGS) involves a drying step in which the temperature at the dryer inlet may be above 500°C, while the temperature at the dryer discharge may be above 100°C [1]. Application of heat to feed ingredients may initiate Maillard reactions, which decrease the concentration and digestibility of Lysine and other amino acids (AA) [2-4]. Lysine is particularly susceptible to undergo Maillard reactions because of its free amino group, which easily reacts with reducing sugars. If the amino group of an AA reacts with a reducing sugar to form early or advanced Maillard reaction products, it becomes unavailable to pigs [2,5]. During the acid hydrolysis step of AA analysis, however, Lysine that has reacted with reducing sugars is partially recovered, thus leading to an overestimation of available Lysine. For this reason, determination of reactive Lysine, color, and the Lysine:CP ratio have been suggested as approaches to estimate the availability of Lysine in DDGS [2,6-9]. Heat damage and Maillard reactions have been described in different sources of corn DDGS [2,10,11]. However, if different sources of DDGS are used, it is difficult to distinguish between effects of heat damage and other factors influencing AA digestibility. Gradual increases in heating of a specific source of DDGS will result in reduced concentrations of reactive Lysine [2,6]. There is, however, no information about effects of increasing time of heating of a specific source of DDGS on *in vivo* AA digestibility, and on the changes in reactive Lysine, and the Lysine:CP ratio. Prediction of the concentration of digestible AA and CP from the concentration of reactive Lysine and the Lysine:CP ratio in corn DDGS that was purposefully heat-damaged have not been reported. Thus, the primary objective of this experiment was to determine effects of heat treatment on the apparent ileal digestibility (AID) and the standardized ileal digestibility (SID) of AA in corn DDGS fed to growing pigs. A second objective was to develop prediction equations that may be used to predict the concentration of SID AA in corn DDGS.

## Materials and methods

The Institutional Animal Care and Use Committee at the University of Illinois reviewed and approved the protocol for this experiment. Pigs used in the experiment were the offspring of G-performer boars and F-25 females (Genetiporc, Alexandria, MN).

### Animals, housing, and experimental design

Ten growing pigs (initial BW: 53.5 ± 3.9 kg) were surgically equipped with a T-cannula in the distal ileum [12] and allotted to a replicated 5 × 4 Youden square design with 5 diets and 4 periods in each square. Pigs were individually housed in a controlled environment in pens (1.2 m ×1.5 m) equipped with a feeder and a nipple waterer.

### Diets and feeding

Distillers dried grains with solubles was obtained from Poet Nutrition (Sioux Falls, SD) and analyzed for CP and AA. The DDGS was divided into 4 batches that were not autoclaved or autoclaved at 130°C for 10, 20, or 30 min (Table 1). Four diets containing DDGS from each of the 4 batches were formulated, with DDGS being the only source of AA and CP in the diets (Tables 2 and 3). A N-free diet also was formulated and used to determine the basal endogenous losses of CP and AA in the pigs. Diets were supplied with vitamins and minerals to meet or exceed the requirement estimates for growing pigs [13]. Chromic oxide also was included (0.4%) in diets and used as an indigestible marker.

**Table 1:** Chemical composition of distillers dried grains with solubles

	**Distillers dried grains with solubles**			
		**Autoclaved at 130°C**		
**Item**	**Non-autoclaved**	**10 min**	**20 min**	**30 min**
Dry matter, %	93.21	92.43	90.91	94.47
Ash, %	5.25	5.08	5.17	5.06
Crude protein (CP), %	27.91	27.44	26.51	27.05
AEE,^1^ %	10.20	12.73	12.83	9.25
Lysine: CP ratio^2^	2.94	2.37	2.75	2.51
Furosine, %	0.015	0.009	0.006	0.008
Reactive Lysine^3^	0.80	0.64	0.72	0.67
Acid detergent fibre, %	7.96	11.05	9.85	10.89
Neutral detergent fibre, %	31.29	33.23	33.32	32.40
Acid detergent lignin, %	0.88	2.06	1.73	2.57
Acid detergent insoluble N,^4^ %	0.12	0.53	0.42	0.55
Reducing sugars, %	0.78	0.60	0.88	0.65
Indispensable AA, %				
Arginine	1.24	1.10	1.19	1.10
Histidine	0.71	0.67	0.70	0.67
Isoleucine	0.97	0.91	0.96	0.93
Leucine	2.92	2.82	2.89	2.78
Lysine	0.82	0.65	0.73	0.68
Methionine	0.53	0.49	0.52	0.50
Phenylalanine	1.24	1.17	1.21	1.17
Threonine	1.02	0.98	1.01	0.98
Tryptophan	0.22	0.20	0.20	0.20
Valine	1.26	1.19	1.26	1.23
Dispensable AA, %				
Alanine	1.90	1.83	1.87	1.82
Aspartic acid	1.75	1.66	1.73	1.66
Cysteine	0.55	0.50	0.52	0.50
Glutamine	4.43	4.31	4.41	4.26
Glycine	1.08	1.04	1.09	1.04
Proline	2.21	2.06	2.13	2.04
Serine	1.31	1.26	1.29	1.23

^1^AEE = acid hydrolyzed ether extract.

^2^Calculated by expressing the concentration of Lysine in each ingredient as a percentage of the concentration of CP [23].

^3^Reactive Lysine (%) = [Lysine (%) – (Furosine (%) ÷ 0.32 × 0.40)]; [2].

^4^*ADIN*, acid detergent insoluble nitrogen.

**Table 2:** Ingredient composition of experimental diets, as-fed basis

	**Distillers dried grains with solubles diets**				
		**Autoclaved at 130°C**			
**Ingredient, %**	**Non-autoclaved**	**10 min**	**20 min**	**30 min**	**N-free diet**
DDGS^1^	60.00	60.00	60.00	60.00	-
Cornstarch	27.00	27.00	27.00	27.00	66.40
Sucrose	10.00	10.00	10.00	10.00	20.00
Solka floc^2^	-	-	-	-	5.00
Soybean oil	-	-	-	-	4.00
Ground limestone	1.20	1.20	1.20	1.20	-
Dicalcium phosphate	0.60	0.60	0.60	0.60	3.00
Sodium chloride	0.40	0.40	0.40	0.40	0.40
Magnesium oxide	-	-	-	-	0.10
Potassium carbonate	-	-	-	-	0.40
Chromic oxide	0.40	0.40	0.40	0.40	0.40
Vitamin-mineral premix^3^	0.40	0.40	0.40	0.40	0.30

^1^*DDGS*, distillers dried grains with solubles.

^2^Fiber Sales and Development Corp., Urbana, OH.

^3^Provided the following per kilogram of complete diet: Vitamin A as retinyl acetate, 11,128 IU; vitamin D_3_ as cholecalciferol, 2,204 IU; vitamin E as DL-alphatocopheryl acetate, 66 IU; vitamin K as menadione nicotinamide bisulfite, 1.42 mg; thiamin as thiamine mononitrate, 0.24 mg; riboflavin, 6.58 mg; pyridoxine as pyridoxine hydrochloride, 0.24 mg; vitamin B_12_, 0.03 mg; D-pantothenic acid as D-calcium pantothenate, 23.5 mg; niacin as nicotinamide, 1.0 mg, and nicotinic acid, 43.0 mg; folic acid, 1.58 mg; biotin, 0.44 mg; Cu, 10 mg as copper sulfate; Fe, 125 mg as iron sulfate; I, 1.26 mg as potassium iodate; Mn, 60 mg as manganese sulfate; Se, 0.3 mg as sodium selenite; and Zn, 100 mg as zinc oxide.

**Table 3:** Analyzed composition of experimental diets, as-fed basis

	**Distillers dried grains with solubles**				
		**Autoclaved at 130°C**			
**Item**	**Non-autoclaved**	**10 min**	**20 min**	**30 min**	**N-free diet**
Dry matter, %	92.42	93.18	92.59	92.32	92.53
Crude protein (CP), %	14.96	15.59	14.62	15.76	0.28
Indispensable AA, %					
Arginine	0.73	0.68	0.70	0.67	-
Histidine	0.42	0.41	0.42	0.41	-
Isoleucine	0.56	0.57	0.58	0.55	-
Leucine	1.69	1.75	1.76	1.72	-
Lysine	0.51	0.42	0.44	0.41	-
Methionine	0.31	0.29	0.29	0.28	-
Phenylalanine	0.71	0.73	0.74	0.72	-
Threonine	0.61	0.61	0.62	0.60	-
Tryptophan	0.13	0.12	0.12	0.12	-
Valine	0.74	0.75	0.76	0.73	-
Dispensable AA, %					
Alanine	1.11	1.14	1.15	0.12	-
Aspartic acid	1.05	1.04	1.06	1.03	-
Cysteine	0.33	0.32	0.31	0.31	-
Glutamic acid	2.60	2.69	2.70	2.65	-
Glycine	0.64	0.65	0.66	0.64	-
Proline	1.30	1.24	1.27	1.23	-
Serine	0.78	0.79	0.79	0.78	-

The amount of feed provided was calculated as 2.5 times the maintenance requirement of energy (i.e., 106 kcal of ME/kg BW^0.75^; [13]). Pigs were fed once daily at 0800 h. At the beginning of each period, feed allowance was adjusted based on the BW of each pig. Water was available at all times.

### Sample collection

Each period consisted of 7 d. The initial 5 d were considered an adaptation period to the diet. On d 6 and d 7, ileal digesta were collected for 8 h using standard operating procedures. A plastic bag was attached to the cannula barrel and digesta flowing into the bag were collected. Bags were replaced whenever they were filled with digesta, or at least once every 30 min and immediately frozen at – 20°C to prevent bacterial degradation of AA in the digesta.

### Chemical analyses

At the conclusion of the experiment, ileal digesta samples were thawed, mixed within animal and diet, and a sub-sample was lyophilized, ground to pass a 1 mm screen, and analyzed. A sample of each diet and of each batch of DDGS was collected at the time of diet mixing. Diets, ingredients, and ileal samples were analyzed for AA by ion-exchange chromatography with postcolumn derivatization with ninhydrin. Amino acids were oxidized with performic acid, which was neutralized with Na metabisulfite [14,15]. Amino acids were liberated from the protein by hydrolysis with 6 mol/L HCL for 24 h at 110°C and quantified with the internal standard by measuring the absorption of reaction products with ninhydrin at 570 nm. Tryptophan was determined by HPLC with fluorescence detection (extinction 280 nm, emission 356 nm), after alkaline hydrolysis with barium hydroxide octahydrate for 20 h at 110°C [16]. Diets, ingredients, and ileal samples also were analyzed for DM (method 935.29; [17]), and for CP following the Dumas procedure (method 968.06; [17]). Diets and ileal samples were analyzed for chromium (method 990.08; [17]). Ingredients were analyzed for acid detergent fibre (ADF; Method 973.18; [17]), neutral detergent fibre (NDF) [18], lignin (method 973.18 (A-D); [17]), ash (method 942.05; [17]), for total fat by acid hydrolysis using 3 mol/L HCl [19] followed by crude fat extraction with petroleum ether (method 2003.06; [17]), total reducing sugars [20], and furosine [8]. The concentration of acid detergent insoluble nitrogen (ADIN) in ingredients was determined as the concentration of N in the ADF fraction (method 990.03; [17]).

### Calculations and statistical analysis

Values for AID and SID of CP and AA were calculated [21]. The Lysine:CP ratio in each DDGS sample was calculated by expressing the concentration of Lysine in the sample as a percentage of the CP in the sample. The concentration of reactive Lysine (%) was calculated by the following equation:

$$\begin{array}{l} \text{Reactive}\;\text{Lysine}=\text{total}\;\text{Lysine}\;\left(\%\right)\\ \;–\left[\text{furosine}\;\left(\%\right)÷0.32\times 0.40\right]; \end{array}$$

Data were analyzed using the MIXED procedure (SAS Institute Inc., Cary, NC). Normality of the data and the presence of outliers were evaluated using the UNIVARIATE procedure of SAS. One outlier was identified and removed from the data. The model included dietary treatment as fixed effect and pig and period as random effects. Linear and quadratic effects of increasing time of heat treatment on the AID and SID of AA were analyzed by orthogonal polynomial contrasts. Correlations among predictor variables or between predictor variables and dependent variables were determined using the CORR procedure of SAS. The coefficients of correlation (r_p_) were divided into 4 groups: no correlation (*P* > 0.05), low correlation (r_p_ < 0.30), moderate correlation (0.60 > r_p_ ≥ 0.30), or high correlation (r_p_ ≥ 0.60). Regression equations to estimate the relationship between the concentration of SID AA and nutrient concentration were developed using the REG procedure in SAS. The pig was the experimental unit for all analyses and significance among means was assessed with an α level of 0.05.

## Results

Heat treatment did not change the concentrations of DM, ash, or CP in DDGS (Table 1). The concentration of total Lysine, however, was 0.82% in non-autoclaved DDGS and 0.65, 0.73, and 0.68% in the DDGS that was autoclaved for 10, 20, and 30 min, respectively. The calculated concentration of reactive Lysine was 0.80, 0.64, 0.72, and 0.67% for non-autoclaved DDGS and DDGS that was autoclaved for 10, 20, and 30 min, respectively. The Lysine:CP ratio was 2.94 in non-autoclaved DDGS, whereas the Lysine:CP ratio was 2.37, 2.75, and 2.51 in the batches that were autoclaved for 10, 20, and 30 min, respectively. The ADF concentration was 7.96% in non-autoclaved DDGS, whereas for autoclaved DDGS, the concentration of ADF was 11.05, 9.85, and 10.89% (10, 20, and 30 min, respectively). The concentration of ADIN in non-autoclaved DDGS was 0.12%, whereas the concentration of ADIN ranged from 0.42 to 0.55% in the autoclaved DDGS. Reducing sugar concentration was 0.78 in non-autoclaved DDGS, but DDGS that was autoclaved for 10, 20, and 30 min contained 0.60, 0.88, and 0.65% reducing sugars, respectively.

### Digestibility of CP and AA

The AID of CP decreased (linear, *P* < 0.05) from 64.4% in the non-autoclaved DDGS to 59.0, 52.2, and 55.7% in the DDGS that was autoclaved for 10, 20, or 30 min, respectively (Table 4). The AID of Ile, Lysine, Methionine, Phenylalanine, and Aspartic acid was reduced (quadratic, *P* < 0.05) with increasing time of autoclaving, whereas the AID of all other AA was reduced (linear, *P* < 0.05) with increasing time of autoclaving. Lysine was the AA most affected by increasing time of autoclaving. The AID of Lysine was reduced (quadratic, *P* < 0.05) from 61.2 to 48.0, 48.7, and 44.9% for the non-autoclaved DDGS and the DDGS that was autoclaved for 10, 20, or 30 min, respectively. The mean AID of indispensable AA was reduced (linear, *P* < 0.05) as time of autoclaving increased.

**Table 4:** **Apparent ileal digestibility of crude protein (CP) and AA in distillers dried grains with solubles subjected to increasing levels of heat treatment by weanling pigs**^**1**^

	**Distillers dried grains with solubles**						
		**Autoclaved at 130°C**			**SEM**	***P*-value**^**2**^	
**Item**	**Non-autoclaved**	**10 min**	**20 min**	**30 min**		**Linear**	**Quadratic**
CP, %	64.4	59.0	52.2	55.7	2.0	< 0.01	0.12
Indispensable AA, %							
Arginine	79.3	71.9	72.0	69.1	1.7	< 0.01	0.06
Histidine	73.9	66.8	65.9	66.0	1.3	< 0.01	0.23
Isoleucine	72.3	66.2	66.6	63.1	1.4	< 0.01	0.02
Leucine	84.0	80.7	80.4	79.1	0.8	< 0.01	0.12
Lysine	61.2	48.0	48.7	44.9	1.9	< 0.01	0.02
Methionine	83.6	77.7	77.8	75.7	0.9	< 0.01	< 0.01
Phenylalanine	79.8	75.1	75.4	73.6	1.0	< 0.01	0.04
Threonine	60.2	53.9	53.2	51.2	1.6	< 0.01	0.27
Tryptophan	55.1	43.7	42.8	41.5	2.0	< 0.01	0.15
Valine	71.9	65.7	65.5	62.6	1.4	< 0.01	0.08
Mean	75.6	70.0	69.9	67.8	1.1	< 0.01	0.07
Dispensable AA, %							
Alanine	77.5	73.3	71.4	71.0	1.3	< 0.01	0.86
Aspartic acid	65.5	55.2	55.4	53.3	1.5	< 0.01	0.02
Cysteine	71.9	64.7	60.5	62.7	1.5	< 0.01	0.54
Glutamic acid	81.7	76.7	75.6	75.3	0.9	< 0.01	0.34
Glycine	43.4	36.3	29.0	31.7	4.6	< 0.01	0.47
Proline	69.6	65.1	64.1	63.2	1.3	< 0.01	0.47
Mean	68.3	61.8	59.2	59.5	1.5	< 0.01	0.85

^1^Data are means of 8 observations, except for the distillers dried grains with solubles diet that was autoclaved for 10 min (n = 7).

^2^Linear and quadratic effects of time of autoclaving.

The SID of CP decreased (linear, *P* < 0.05) from 77.9% in the non-autoclaved DDGS to 72.1, 66.1, and 68.5% in the DDGS that was autoclaved for 10, 20, or 30 min, respectively (Table 5). The SID of Lysine was reduced (quadratic, *P* < 0.05) from 66.8% in the non-autoclaved DDGS to 54.9, 55.3, and 51.9% in the DDGS that was autoclaved for 10, 20, or 30 min, respectively.

**Table 5:** **Standardized ileal digestibility of crude protein (CP) and AA in distillers dried grains with solubles subjected to increasing levels of heat treatment by weanling pigs**^**1**^

	**Distillers dried grains with solubles**						
		**Autoclaved at 130°C**			**SEM**	***P*-value**^**2**^	
**Item**	**Non-autoclaved**	**10 min**	**20 min**	**30 min**		**Linear**	**Quadratic**
CP, %	77.9	72.1	66.1	68.5	2.0	< 0.01	0.25
Indispensable AA, %							
Arginine	87.9	81.3	81.0	78.5	1.7	< 0.01	0.11
Histidine	78.1	71.2	70.1	70.3	1.3	< 0.01	0.27
Isoleucine	77.3	71.1	71.4	68.2	1.4	< 0.01	0.03
Leucine	86.7	83.3	83.1	81.8	0.8	< 0.01	0.12
Lysine	66.8	54.9	55.3	51.9	1.9	< 0.01	0.04
Methionine	86.1	80.4	80.5	78.5	0.9	< 0.01	< 0.01
Phenylalanine	83.7	79.0	79.2	77.5	1.0	< 0.01	0.05
Threonine	70.2	64.0	63.1	61.3	1.6	< 0.01	0.33
Tryptophan	65.9	55.5	54.5	53.2	2.0	< 0.01	0.20
Valine	77.2	71.0	70.7	67.9	1.4	< 0.01	0.09
Mean	80.7	75.2	74.9	73.1	1.1	< 0.01	0.09
Dispensable AA, %							
Alanine	83.3	79.1	77.1	76.8	1.3	< 0.01	0.89
Aspartic acid	72.3	62.1	62.1	60.2	1.5	< 0.01	0.03
Cysteine	77.7	70.8	66.8	68.9	1.5	< 0.01	0.55
Glutamic acid	85.2	80.1	79.0	78.7	0.9	< 0.01	0.33
Glycine	73.4	66.0	58.1	61.7	4.6	< 0.01	0.39
Proline	76.9	72.4	71.3	70.5	1.3	< 0.01	0.48
Mean	78.1	71.7	69.0	69.4	1.5	< 0.01	0.91

^1^Data are means of 8 observations, except for the distillers dried grains with solubles diet that was autoclaved for 10 min (n = 7); Values for standardized ileal digestibility were calculated by correcting apparent ileal digestibility values for basal endogenous losses (g/kg of DMI), which were determined by feeding pigs a N-free diet: CP, 21.93; Arginine, 0.68; Histidine, 0.19; Isoleucine, 0.30; Leucine, 0.50; Lysine, 0.31; Methionine, 0.08; Phenylalanine, 0.30; Threonine, 0.66; Tryptophan, 0.15; Valine, 0.43; Alanine, 0.71; Aspartic acid, 0.77; Cysteine, 0.21; Glutamic acid, 0.98; Glycine, 2.08; and Serine, 0.62.

^2^Linear and quadratic effects of time of autoclaving.

The SID of Isoleucine, Methionine, Phenylalanine, and Aspartic acid also was reduced (quadratic, *P* < 0.05) with increasing time of autoclaving, whereas the SID of all other AA was reduced (linear, *P* < 0.05) with increasing time of autoclaving. The mean SID of indispensable AA also was reduced (linear, *P* < 0.05) as time of autoclaving increased.

### Coefficients of linear correlation and regression equations

The concentrations of SID AA were correlated (r_p_ > 0.70; *P* < 0.01) with the Lysine:CP ratio, except for the concentration of SID Threonine, which was only moderately correlated with the Lysine:CP ratio (Table 6). The concentration of SID AA was poorly correlated (r_p_ < 0.50) with the concentration of reducing sugars. The concentrations of SID AA were negatively correlated with the concentrations of ADF, NDF, lignin, and ADIN (*P* < 0.01). The concentration of SID of each AA also was well correlated (r_p_ > 0.65; *P* < 0.01) with its respective concentration of total AA, except for SID Threonine, which was only moderately correlated (r_p_ < 0.65; *P* < 0.01) with the concentration of total Threonine.

**Table 6:** **Coefficients of linear correlation between nutrient composition of distillers dried grains with solubles and the concentration (%) of standardized ileal digestible (SID) AA**^**1**^

	**Nutrient composition**															
	**Lys:CP**	**Reducing sugars**	**ADF**	**NDF**	**Lignin**	**ADIN**^**2**^	**Arg**	**His**	**Ile**	**Leu**	**Lys**	**Met**	**Phe**	**Thr**	**Trp**	**Val**
**Item**																
SID Arg	0.80^*^	0.50^*^	-0.86^*^	-0.60^*^	-0.62^*^	-0.87^*^	0.84^*^	-	-	-	-	-	-	-	-	-
SID His	0.72^*^	0.06^ns^	-0.81^*^	-0.69^*^	-0.48^*^	-0.82^*^	-	0.69^*^	-	-	-	-	-	-	-	-
SID Ile	0.76^*^	0.49^*^	-0.81^*^	-0.58^*^	-0.55^*^	-0.81^*^	-	-	0.70^*^	-	-	-	-	-	-	-
SID Leu	0.72^*^	0.45^**^	-0.81^*^	-0.54^*^	-0.65^*^	-0.82^*^	-	-	-	0.78^*^	-	-	-	-	-	-
SID Lys	0.83^*^	0.45^**^	-0.91^*^	-0.74^*^	-0.54^*^	-0.92^*^	-	-	-	-	0.89^*^	-	-	-	-	-
SID Met	0.78^*^	0.40^**^	-0.86^*^	-0.73^*^	-0.48^*^	-0.87^*^	-	-	-	-	-	0.75^*^	-	-	-	-
SID Phe	0.78^*^	0.47^*^	-0.85^*^	-0.63^*^	-0.56^*^	-0.86^*^	-	-	-	-	-	-	0.83^*^	-	-	-
SID Thr	0.58^*^	0.25^ns^	-0.70^*^	-0.55^*^	-0.56^*^	-0.73^*^	-	-	-	-	-	-	-	0.60^*^	-	-
SID Trp	0.71^*^	0.34^ns^	-0.81^*^	-0.70^*^	-0.52^*^	-0.83^*^	-	-	-	-	-	-	-	-	0.84^*^	-
SID Val	0.73^*^	0.45^**^	-0.78^*^	-0.59^*^	-0.50^*^	-0.78^*^	-	-	-	-	-	-	-	-	-	0.55^*^

^1^*Arg*, Arginine; *His*, Histidine; *Ile*, Isoleucine; *Leu*, Leucine; *Lys*, Lysine; *Met*, Methionine; *Phe*, Phenylalanine; *Thr*, Threonine; *Trp*, Tryptophan; *Val*, Valine.

^2^*ADIN*, acid detergent insoluble nitrogen.

^*^= *P* < 0.01; ^**^= *P* < 0.05; ns = not significant.

The concentrations of digestible AA may be predicted by regression equations presented in Table 7. The concentration of SID Arginine or SID Lysine were best predicted by equations that included the concentration of ADIN in the model (r^2^ = 0.76 and 0.84, respectively). The concentration of most SID AA was predicted (r^2^ > 0.50) by the concentration of each total AA, but the concentration of SID Threonine was poorly (r^2^ = 0.36) predicted by the concentration of Threonine.

**Table 7:** **Linear regression to predict the concentration (%) of standardized ileal digestible (SID) AA from the nutrient concentrations (%) in corn distillers dried grains with solubles (DDGS) fed to pigs**^**1**^

	**Intercept**			**Independent variable**^**2**^				
**Dependent variable**	**Estimate**	**SE**	***P*-value**	**Variable**	**Estimate**	**SE**	***P*-value**	**r**^**2**^
SID Arginine	-0.66	0.20	< 0.01	Arg	1.398	0.171	< 0.01	0.70
	1.16	0.02	< 0.01	ADIN	-0.506	0.053	< 0.01	0.76
SID Histidine	-0.62	0.22	< 0.01	His	1.639	0.322	< 0.01	0.47
	0.58	0.01	< 0.01	ADIN	-0.203	0.025	< 0.01	0.68
SID Isoleucine	-0.92	0.30	< 0.01	Ile	1.706	0.320	< 0.01	0.50
	0.15	0.08	0.08	Lys:CP	0.200	0.031	< 0.01	0.58
SID Leucine	-2.23	0.69	< 0.01	Leu	1.623	0.243	< 0.01	0.61
	2.61	0.03	< 0.01	ADIN	-0.554	0.071	< 0.01	0.67
SID Lysine	-0.46	0.08	< 0.01	Lys	1.214	0.115	< 0.01	0.79
	0.60	0.02	< 0.01	ADIN	-0.463	0.037	< 0.01	0.84
SID Methionine	-0.39	0.13	< 0.01	Met	1.53	0.252	< 0.01	0.56
	0.46	0.01	< 0.01	ADIN	-0.161	0.017	< 0.01	0.76
SID Phenylalanine	-1.11	0.26	< 0.01	Phe	1.726	0.217	< 0.01	0.68
	1.08	0.01	< 0.01	ADIN	-0.304	0.034	< 0.01	0.73
SID Threonine	-1.31	0.50	< 0.05	Thr	1.967	0.499	< 0.01	0.36
	0.75	0.02	< 0.01	ADIN	-0.247	0.043	< 0.01	0.54
SID Tryptophan	-0.24	0.04	< 0.01	Trp	1.767	0.219	< 0.01	0.70
	-0.04	0.03	0.19	Lys:CP	0.061	0.011	< 0.01	0.51
SID Valine	-0.88	0.50	0.08	Val	1.438	0.400	< 0.01	0.31
	0.24	0.11	0.04	Lys:CP	0.246	0.042	< 0.01	0.54

^1^n = 31 observations; for all models *P* < 0.01.

^2^*ADIN*, acid detergent insoluble nitrogen; *Arg*, Arginine; *His*, Histidine; *Ile*, Isoleucine; *Leu*, Leucine; *Lys*, Lysine; *Met*, Methionine; *Phe*, Phenylalanine; *Thr*, Threonine; *Trp*, Tryptophan; *Val*, Valine.

## Discussion

### Composition of distillers dried grains with solubles

The concentrations of DM, ash, and CP were not affected by autoclaving DDGS and this observation supports results of González-Vega et al. [4] who reported that autoclaving soybean meal did not change concentrations of DM, ash, or CP. Changes in the concentration of Lysine observed for autoclaved DDGS in this experiment also agree with results of previous experiments in which the concentration of Lysine was decreased by heat treatment [2,3,6,22]. Based on the concentration of Lysine, these results indicate that autoclaving corn DDGS for 10 min was sufficient to cause heat damage, but increasing time of autoclaving to 20 and 30 min did not result in further decreases in Lysine concentration. In contrast, in a previous experiment, the concentration of Lysine in corn DDGS autoclaved for 0, 10, 20, or 30 min at 135°C was linearly decreased from 0.82 to 0.59% [6]. These differences may be a result of differences in sample preparation and autoclaving procedures. Although temperature and time of autoclaving were similar between the 2 experiments, in this experiment, DDGS was autoclaved on an as-is basis in quantities of 2.5 kg per tray whereas Fontaine et al. [6] ground DDGS to < 3 mm particle size and then autoclaved DDGS in 250 g quantities. The concentrations of furosine in DDGS used in this experiment were slightly less than the average concentration of furosine (0.02%; CV = 91.4%) measured in 21 sources of DDGS, and these differences may be a result of the high degree of variation when determining the concentration of furosine in DDGS [8]. The reason for the reduced Lysine:CP ratio in the autoclaved DDGS is that only the concentration of Lysine, but not the concentration of CP, is reduced when heat damage occurs [8,23]. The Lysine:CP ratio also was reduced in soybean meal that was heat-damaged compared with unheated soybean meal [4]. The concentration of Lysine in the non-autoclaved DDGS was slightly greater than the average concentration of Lysine (0.76%) in 39 sources of DDGS [24]. Likewise, the Lysine:CP ratio was slightly greater in the unheated DDGS compared with the average value (2.77) reported previously [24]. However, the concentrations of Lysine and the Lysine:CP ratio in autoclaved DDGS are close to the average values reported by Stein and Shurson [24] for corn DDGS, which indicates that the heat damage caused by autoclaving simulated the varying degrees of heat damage caused by processing of DDGS in commercial production facilities.

The concentration of ADIN has been used as a predictor of heat damage in plant proteins and our results agree with these observations [25]. The changes observed between the concentrations of reducing sugars in the unheated DDGS and the 3 autoclaved DDGS were expected because, in the initial steps of the Maillard reactions, reducing sugars react with the ϵ-amino group of Lysine to form fructoselysine and maltuloselysine, respectively [26].

### Digestibility of CP and AA

Values for the AID of CP and AA in the DDGS that was not autoclaved are in agreement with results from previous experiments [11,24]. Results of previous experiments also indicated that the digestibility of Lysine was more reduced by heat damage than the digestibility of other AA [4,22]. Values for the SID of CP and AA in the non-autoclaved DDGS were within the range of values for the SID of CP and AA in corn DDGS observed in previous experiments [24]. This observation is supported by the value for the Lysine:CP ratio (2.94) determined for corn DDGS in this experiment, which is in agreement with the average value (2.77) reported previously [24]. For the autoclaved DDGS, however, values for the SID of CP and AA were less than those reported by Stein and Shurson [24]. Reductions observed for the SID of Lysine with increasing time of autoclaving and the results from reducing sugars and the Lysine concentrations in autoclaved DDGS indicate that the conditions created by autoclaving of DDGS (i.e., heat, moisture, and pressure) were favorable to initiation of Maillard reactions, which renders Lysine unavailable to the animals and, therefore, reduces Lysine digestibility [4,27]. This indicates that heating alone may not be detrimental to the nutritional quality of DDGS, unless combined with a certain degree of moisture. The nutritional quality of soybean meal heat-processed in conventional oven was not different from that of conventional soybean meal, which is in agreement with the latter assumption [4]. It appears, however, that the degree of heat damage among autoclaved DDGS (i.e., 10, 20, or 30 min) was not different and this observation is supported by the fact that the SID of Lysine among these ingredients was not different. Reductions in the digestibility of AA other than Lysine have been attributed to the formation of cross-linkages between protein chains [28]. When these cross-links are formed, digestive enzymes such as trypsin have reduced access to the proteins, thus the ability of trypsin to hydrolyze peptide bonds is reduced. Exposure to excessive heat and pressure may also lead to AA racemization, thus converting L-AA to D-AA [29]. Proteolytic enzymes are unable to hydrolyze peptide bonds connecting D-AA and L-AA. Consequently, the digestibility is reduced not only for the D-AA, but also for the L-AA [30].

### Coefficients of linear correlation and regression equations

The relatively good correlations between the concentration of SID AA and the concentration of each AA in DDGS agree with previous results [8]. Consequently, analyzed concentrations of most AA in DDGS may be used to predict the concentration of SID AA in DDGS although that is not the case for Threonine. It has been observed that heat damage of feed ingredients is associated with an increase in analyzed values for ADF, lignin, and ADIN, which results in a decrease in N digestibility in cattle and sheep [30]. The concentrations of ADF and ADIN is increased in dark-colored DDGS, which indicates a greater degree of heat damage in such ingredients [10], and likely a lower digestibility of AA. The negative correlation between the concentration of SID AA and the concentrations of ADF, lignin, or ADIN observed in this experiment support the latter observations.

The concentration of SID Lysine can accurately be predicted from the concentration of Lysine. This observation agrees with Kim et al. [8]. Prediction of the concentration of SID Lysine in this study was slightly improved if the concentration of ADIN was used in the model. This indicates that the concentration of analyzed ADIN may be used to predict the concentration of SID Lysine in DDGS.

## Conclusions

Results of this experiment confirmed that the concentration and digestibility of AA in DDGS is reduced as a result of heat damage. The concentrations of Lysine and ADIN are good predictors of the concentration of most AA in heat-damaged DDGS, which indicates that such predictors may be used as indicators of the protein quality in DDGS. These equations, however, must be validated using different sources of DDGS.

## Abbreviations

AA: Amino acids; ADF: Acid detergent fibre; ADIN: Acid detergent insoluble nitrogen; AID: Apparent ileal digestibility; BW: Body weight; CDS: Condensed solubles; CP: Crude protein; CV: Coefficient of variation; DDGS: Distillers dried grains with solubles; DM: Dry matter; HPLC: High performance liquid chromatography; ME: Metabolizable energy; NDF: Neutral detergent fibre; SID: Standardized ileal digestibility.

## Competing interests

The authors declare that they have no competing interests.

## Authors’ contributions

All authors read and approved the final manuscript.
